# Cyanobacteria and Cyanotoxins: The Influence of Nitrogen versus Phosphorus

**DOI:** 10.1371/journal.pone.0038757

**Published:** 2012-06-15

**Authors:** Andrew M. Dolman, Jacqueline Rücker, Frances R. Pick, Jutta Fastner, Thomas Rohrlack, Ute Mischke, Claudia Wiedner

**Affiliations:** 1 Chair of Freshwater Conservation, Brandenburg University of Technology, Bad Saarow, Germany; 2 Department of Biology, University of Ottawa, Ottawa, Canada; 3 Federal Environmental Agency, Berlin, Germany; 4 Department of Plant and Environmental Sciences, Norwegian University of Life Sciences, Ås, Norway; 5 Department of Shallow Lakes and Lowland Rivers, Leibniz-Institute of Freshwater Ecology and Inland Fisheries (IGB), Berlin, Germany; Uppsala University, Sweden

## Abstract

The importance of nitrogen (N) versus phosphorus (P) in explaining total cyanobacterial biovolume, the biovolume of specific cyanobacterial taxa, and the incidence of cyanotoxins was determined for 102 north German lakes, using methods to separate the effects of joint variation in N and P concentration from those of differential variation in N versus P. While the positive relationship between total cyanobacteria biovolume and P concentration disappeared at high P concentrations, cyanobacteria biovolume increased continually with N concentration, indicating potential N limitation in highly P enriched lakes. The biovolumes of all cyanobacterial taxa were higher in lakes with above average joint NP concentrations, although the relative biovolumes of some Nostocales were higher in less enriched lakes. Taxa were found to have diverse responses to differential N versus P concentration, and the differences between taxa were not consistent with the hypothesis that potentially N_2_-fixing Nostocales taxa would be favoured in low N relative to P conditions. In particular *Aphanizomenon gracile* and the subtropical invasive species *Cylindrospermopsis raciborskii* often reached their highest biovolumes in lakes with high nitrogen relative to phosphorus concentration. Concentrations of all cyanotoxin groups increased with increasing TP and TN, congruent with the biovolumes of their likely producers. Microcystin concentration was strongly correlated with the biovolume of *Planktothrix agardhii* but concentrations of anatoxin, cylindrospermopsin and paralytic shellfish poison were not strongly related to any individual taxa. Cyanobacteria should not be treated as a single group when considering the potential effects of changes in nutrient loading on phytoplankton community structure and neither should the N_2_-fixing Nostocales. This is of particular importance when considering the occurrence of cyanotoxins, as the two most abundant potentially toxin producing Nostocales in our study were found in lakes with high N relative to P enrichment.

## Introduction

Anthropogenic loading of nitrogen (N) and phosphorus (P) to freshwaters and coastal marine systems, is a global environmental problem [1], [2] that generates social and financial costs for human populations [3], [4]. One of the more unpleasant consequences of eutrophication is an increase in the occurrence of unsightly, odorous, and sometimes toxic cyanobacterial blooms [5], [6]. Control of cyanobacteria is thus a major concern in freshwater management.

The observation that cyanobacteria increase with eutrophication has been recognized for several decades [7]–[12]. For a long time phosphorus was considered the primary nutrient limiting the development of cyanobacterial biovolumes [13], [14] despite there being considerable evidence that N limitation is a common and widespread phenomenon in lakes [15], [16]. Nitrogen consequently received less attention than phosphorus and there is less known about the role nitrogen may play in controlling eutrophication, and in particular its influence on the taxonomic composition of phytoplankton [17], [18].

There has been a great deal of research into the various traits, such as buoyancy regulation and colony formation, that allow cyanobacteria to become dominant in the phytoplankton assemblages of highly eutrophic lakes; reviewed in [19], [20]. One such trait is the ability of some cyanobacteria to fix atmospheric nitrogen (N_2_) giving them a competitive advantage when the ratio of N to P is low and N availability is limiting phytoplankton growth rates [21]. Given the tendency for more eutrophic lakes to have a lower ratio of N to P [22] this has been suggested as one reason why cyanobacteria tend to dominate the phytoplankton in eutrophic lakes [23]. More common is the claim that cyanobacteria become dominant in lakes with a low N to P ratio in general and that reducing nitrogen inputs as a means of controlling phytoplankton would be a waste of effort because cyanobacteria could readily replace the missing nitrogen through fixation [24], [25]. Thus legislation governing inputs to water systems and recommendations to water managers have in the past focused on P reduction.

The cyanobacteria are highly diverse and the traits that may help them to dominate are not shared by all taxa. For example, N_2_ fixation can only be done by the Nostocales, and a small number of non-heterocystous taxa such as the marine *Trichodesmium*. However, most studies examining the response of phytoplankton composition to changes in nutrient concentrations across a large number of lakes treat cyanobacteria as a single response group, e.g. [10]–[12], [26]. Many studies have examined compositional changes in individual lakes during eutrophication (or abatement) e.g. [27]–[29] or have examined the ecology of specific taxa, e.g. [30] but there have been surprisingly few comprehensive studies of how functional groups of cyanobacteria change along eutrophication gradients and fewer still along gradients of both P and N (but see [31]).

The occurrence of particular cyanotoxins is dependent on the composition of cyanobacteria communities because different species can produce different toxins. For example, microcystins are mainly produced by *Microcystis*, *Planktothrix* or *Anabaena* species [32]; cylindrospermopsin by *Cylindrospermopsis* and *Aphanizomenon* [33]; and anatoxin and paralytic shellfish poison by various *Anabaena* and *Aphanizomenon* species [34]–[36]. Furthermore there are some taxa, such as the *Limnothrix*, for which no toxic substances have yet been identified. Additionally, it is known that toxigenic and nontoxigenic strains coexist within populations of the same species and that the proportion of toxigenic and nontoxigenic cells in a population can be quite variable [37], [38]. Total toxin concentrations in the water column also depend on the cellular toxin content of the producing taxa, which can be affected by environmental parameters such as light, temperature and nutrients [39]–[41].

Although there are numerous studies on individual toxins or toxin producers there are no comprehensive studies that provide information about concentrations of different toxins, biovolumes of cyanobacterial taxa, and nutrient concentrations from lakes with different trophic status. Graham et al [42] examined microcystin concentrations across a range of TN and TP concentrations in Midwestern lakes of the USA but did not report the biovolumes of individual cyanobacterial species and did not separate the N and P gradients. Graham et al [43] examined the co-occurrence of a range of toxin types and potential producers in cyanobacterial blooms but did not relate this to nutrient concentrations. Therefore the extent to which concentrations of different toxins can be predicted by the biovolume of their producing species, or more generally by the trophic status of lakes, is still unclear.

Through a between lake analysis of data collected from temperate lakes encompassing a wide trophic gradient, and a wide range of N:P ratios, we examined the response of total cyanobacterial biovolume, and the distribution of 9 cyanobacteria taxa and their associated toxins, along nitrogen and phosphorus concentration gradients, using a statistical method that separates the effects of joint variation in N and P concentration from variation in N versus P concentration. The analysis was restricted to the late summer and early autumn period when cyanobacteria are more likely to dominate and potentially bloom in temperate regions (Sommer et al. 1986), and the recreational use of lakes is highest. We tested the following hypotheses: 1) cyanobacterial taxa differ in their preference for relatively N or P enriched waters; 2) the biovolume of N-fixing cyanobacteria is higher in lakes with relatively lower N enrichment relative to P enrichment; 3) the association between N and P enrichment and cyanotoxin concentrations mirrors that of the toxin producing taxa.

## Materials and Methods

### Sampling and Data Preparation

Data on the total phosphorus (TP) and total nitrogen (TN) concentrations, and the biovolume and composition of phytoplankton in the epilimnion or euphotic zone, were compiled for 102 lakes in the north German state of Brandenburg; table 1 gives an overview of the characteristics of these lakes. Water samples were collected and analysed as part of the sampling programmes of the Chair of Freshwater Conservation, Brandenburg University of Technology, Bad Saarow and the State Office of Environment, Health and Consumer Protection (LUGV), Potsdam. Most lakes were sampled monthly to fortnightly at their deepest point for a period of several years, but this analysis uses only samples collected in late summer (August to September). Mixed samples, from the whole water column in polymictic lakes and of the epilimnion in stratified lakes, were prepared by taking samples at half-meter intervals with a 2.3 L Limnos sampler (Turku, Finland). Cyanobacterial composition and biovolume were analysed using an aliquot of the mixed sample, fixed with Lugol’s solution, and estimated using an inverted microscope [44], [45]. Other aliquots were used to determine concentrations of total phosphorus (TP) and total nitrogen (TN) according to standard methods [46].

**Table 1:** Physical and chemical characteristics of sampled lakes.

	Taxon data set	Toxin data set
Location	Brandenburg, Germany	Berlin and Brandenburg, Germany
Number of lake–summers	182	CYN 56; ATX 38; MC 29; PSP 25
Number of unique lakes	102	CYN 30; ATX 14; MC & PSP 13
Summers per lake	1 from 87 lakes; 2–5 from6 lakes; 6–9 from 6 lakes;10–12 from 3 lakes	
Median and range of meandepth	4.85 m (0.98–23.5)	
Median and range of TN	907 µg l^−1^ (215–2500)	996 µg l^−1^ (282–2855)
Median and range of TP	42.3 µg l^−1^ (5.0–354)	42.6 µg l^−1^ (5.8–131)
OECD (1982) TP basedtrophic classes.	Oligo- 4; Meso- 68; Eutro- 87;Hypertrophic- 23 lake summers	
Median and range of the TN:TP ratio	21.8 (2.8–96)	22.1 (11.5–92)
Median and range of Chlorophyll-a	28.7 µg l^−1^ (1–193)	
OECD (1982) chlorophyll–abased trophic classes.	Oligo- 8; Meso- 19; Eutro- 52;Hypertrophic 99 lake summers	

Nine cyanobacterial taxa were identified from these data that we could be confident were unambiguously identified and which occur often enough in the region to be meaningfully analysed. These were *Planktothrix agardhii* (*Planktothrix rubescens* was very rare); the fine filamentous Oscillatoriales of the genera *Pseudanabaena, Limnothrix,* and *Planktolyngbya*, which were grouped because unambiguous taxonomic separation of individual species was not always possible (herein referred to as FFO); three Nostocales of the genera *Aphanizomenon:* (*A. gracile*, *A. issatschenkoi* and *A. flos-aquae*); *Cylindrospermopsis raciborskii*; the genus *Anabaena* (16 species: mainly *Anabaena flos-aquae, Anabaena viguieri* and *Anabaena macrospora*); and *Anabaenopsis* (*Anabaenopsis elenkinii* and *Anabaenopsis cunningtonii*); and finally *Microcystis* of the order Chroococcales (7 species: mainly *Microcystis wesenbergii, Microcystis flos-aquae* and *Microcystis aeruginosa*). Table 2 gives a summary of the occurrence of these taxa in the data set.

**Table 2:** Frequency of occurrence and contribution to total cyanobacteria biovolume of cyanobacterial taxa.

Taxon	No. lake–summers	% of cyanobacteria biovolume	Maximum biovolume mm^3^ l^−1^
FFO	157	50.75	29.6
*A.gracile*	135	13.15	7.46
*Anabaena*	127	3.73	5.36
*P.agardhii*	127	22.65	36.2
*Microcystis*	97	3.61	8.61
*A.issatschenkoi*	91	1.61	1.54
*C.raciborskii*	91	3.3	5.40
*A.flos-aquae*	66	1.04	2.34
*Anabaenopsis*	33	0.15	0.389

Seven lakes were excluded from the analysis because their TN or TP concentrations were so large that they remained outliers even after log_10_ transformation and would therefore represent a poorly sampled set of environmental conditions. A further 22 out of 365 samples were eliminated where biovolume estimates for one or more taxa were extremely high and many times higher than estimates from the preceding and subsequent visits to the same lake; these were probably due to the collection of a wind aggregated scum.

For a subset of the Brandenburg lakes plus some Berlin lakes, the concentrations of four groups of cyanobacterial toxins were measured: microcystin (MC as total microcystins), cylindrospermopsin (CYN), anatoxin-a (ATX), and paralytic shellfish poisons (PSP). Toxin data were collected during the course of several projects over different years and details of the procedures varied a little between projects. In brief, aliquots of mixed water samples were filtered and the dissolved and particulate fractions of CYN, ATX and PSP were determined from the filtrate and the retentate respectively; for MC only the particulate fraction was determined because the dissolved fraction is typically negligible [47].

Microcystin analysis 1995–1996 and 2007–2009: samples were extracted with 75% aqueous methanol and the crude extract analysed for microcystins by High Performance Liquid Chromatography with Photodiode Array Detection (HPLC-PDA) as described in detail here [48], [49]; validation of these methods has been published previously [50]. The detection limit was 1 ng microcystin on column.

Microcystin analysis 1995–1996: samples were taken with a plankton net and microcystin concentrations were calculated per dry weight of collected material. Microcystin concentrations per liter were then calculated using a measurement of seston dry weight per liter determined by filtration of lake water on glass fibre filters and drying at 105°C.

Microcystin analysis 2005–2006: microcystins were extracted from filters of field samples after lyophilisation using 50% methanol. The quantification was done by liquid chromatography with mass spectrometric detection (LC-MS/MS) according to [51]. Microcystins LR, RR and YR, purchased from Sigma-Aldrich Norway A/S (Oslo, Norway), served as a standard to calibrate the system.

CYN, ATX, PSP analysis 2007–2009: samples were extracted with acetonitrile-water-formic acid (75:14.9:0.1) and the crude extract analysed by Liquid Chromatography-Tandem Mass Spectrometry (LC-MS/MS); described in detail in [35], [52]. The detection limit for CYN and ATX was 1 pg on column and for PSPs congener specific between 1–30 pg on column.

ATX analysis 1995–1996: analysis was performed by Gas Chromatography Mass Spectrometry (GC-MS) after derivatization of anatoxin-a as described in detail in [53].

### Data Analysis

We first used nonparametric quantile regression to model total cyanobacterial biovolume as a function of total nitrogen (TN) and total phosphorus (TP) concentrations. Quantile regression differs from ordinary-least-squares (OLS) regression in that rather than modelling the expected mean value of a response variable as a function of predictor variables, one or more quantiles of a response variable are modelled. Using the 50% quantile corresponds to modelling the expected median value, i.e. the value above and below which 50% of observations are expected to fall, while a 90% quantile regression line indicates for a given value of the predictor variable the value of the response variable below which 90% of observations are expected to fall. Quantile regression is useful where there are unknown or unmeasured factors that limit the response variable in addition to those being used as predictors in the model [54]. Modelling a quantile at the upper end of the range can be viewed as asking the question: “what value does the response variable attain when other (possibly unknown) factors are not limiting”. Here we used the 90% quantile to identify ranges of the TN and TP concentrations where maximum biovolumes of cyanobacteria were found. The 90% quantile gave a good compromise between identifying the location of the very largest biovolumes, which may be strongly influenced by outliers, and detecting no pattern in the biovolumes of rarer taxa due to a large number of zero biovolume estimates.

**Figure 1:**
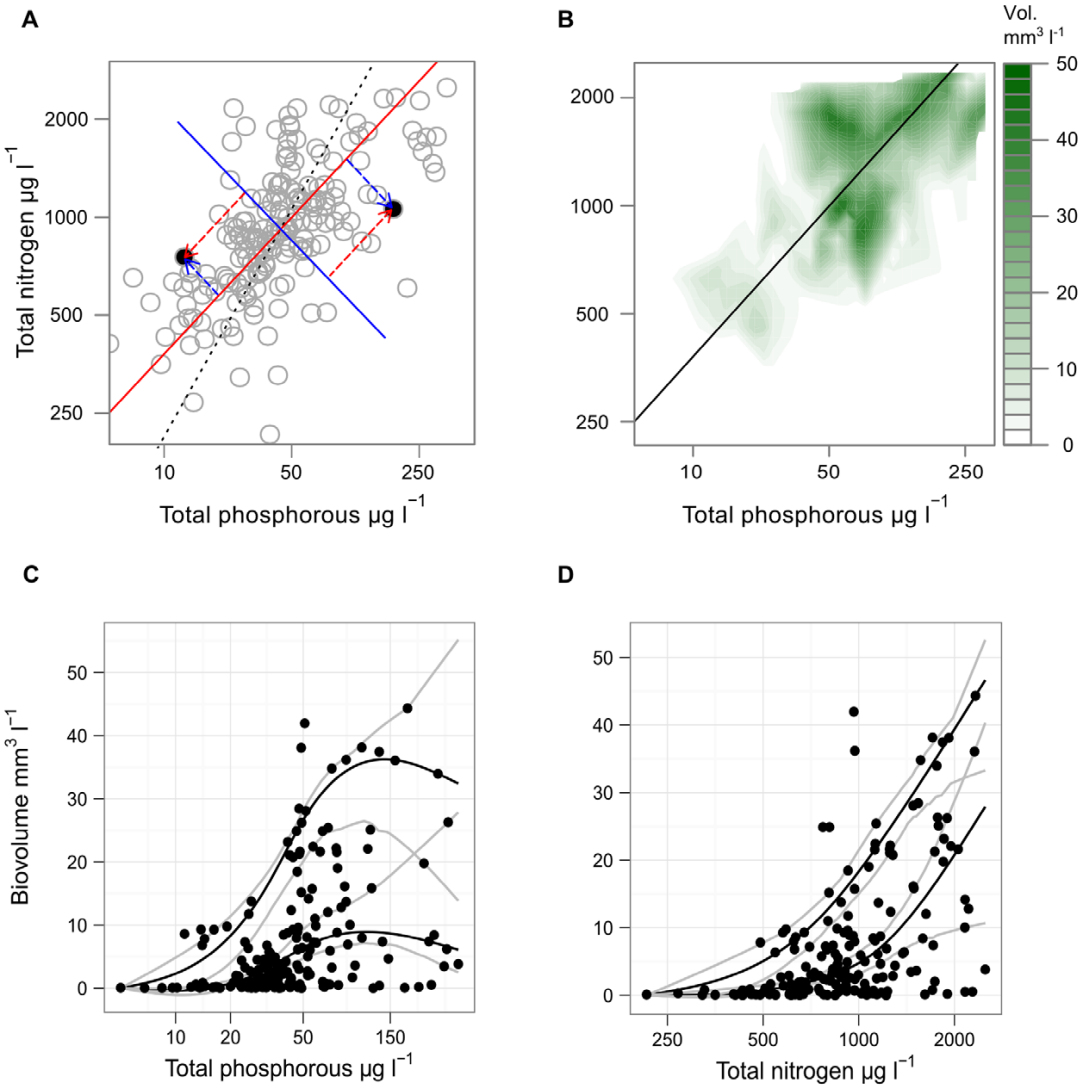
Relationships between TN, TP and total cyanobacterial biovolume. (A) the relationship between total phosphorus and total nitrogen, with a fitted standardised-major-axis (solid red line), the corresponding minor axis (solid blue line), and an isoline (dotted black) indicating points where the TN:TP ratio is equal to the average for the data set. The red arrows illustrate how a point’s joint NP enrichment score is defined as its position on the standardised-major-axis between TN and TP, while the blue arrow shows how its relative TN vs. TP enrichment score is defined as its position on the minor axis. (B) a filled contour plot indicating the fitted 90% quantile of total cyanobacteria biovolume as an estimate of the maximum expected biovolume at combinations of TN and TP concentration. (C) the relationship between cyanobacterial biovolume and total phosphorus and (D) total nitrogen concentration. Fitted lines are natural splines with 4 degrees of freedom showing the 90% and 50% quantiles of observations as a function of TP and TN. Splines were forced through the origin corresponding to an assumption of zero biovolume at zero nutrient concentrations.

In order to separate the effect of variation in N and P concentrations relative to each other from the general response of cyanobacteria to joint increases in both N and P, we analysed the occurrence of cyanobacteria along two derived axes. Standardised-major-axis regression (SMA) between the correlated TN and TP measurements was used to derive, for each lake-summer, a score measuring its position on an axis of joint nitrogen and phosphorus enrichment and an uncorrelated score measuring its position on an orthogonal axis that indicates relative nitrogen versus phosphorus enrichment. The standardised-major-axis describes the structural relationship between two (standardised) variables. Fitted values measure the distance of a point along the fitted line from the origin as a function of both variables and it is these fitted values that we used as a score of joint nitrogen and phosphorus enrichment. Residuals are measured and minimized perpendicularly to the fitted line, rather than vertically to the line as in ordinary-least-squares regression [55], and we used these distances to measure relative nitrogen versus phosphorus enrichment (see Figure 1a).

These axes were used to measure a taxon’s tendency to be located in relatively nutrient rich or poor lakes (its position along the joint enrichment axis) and also its tendency to be located in lakes enriched relatively more by nitrogen than by phosphorus (its position on the relative N vs. P axis). An estimate was made of the median position of each taxon along each of the two axes; this was achieved by calculating a cumulative sum of its biovolume along an axis and identifying the point at which 50% of the biovolume had been reached. In order to adjust for the uneven sampling effort along the joint enrichment and N vs. P axes, which would bias the location of taxa toward the centre of both axes, biovolumes were inversely weighted by the density of sampling points at their corresponding point on the axis being used. Bias-corrected and accelerated 95% confidence intervals of these median estimates were constructed by bootstrapping using the ‘boot’ package for R with 10,000 re-samplings [56].

Filled contour plots were used to visualise the maximum biovolume of total cyanobacteria and of 9 cyanobacteria taxa on both the TN and TP gradients simultaneously. Natural splines of the 90% quantiles of biovolumes were fit, using the R package quantreg [57], to nutrient enrichment and N vs. P enrichment axis scores derived from the raw TN and TP concentrations (see above) as this removed the problem of collinearity between the predictors. The resulting fitted values were then used to interpolate a response surface that was plotted on the original TN and TP axes. Fitted splines had a high degree of complexity, up to 9 degrees of freedom per variable, and the resulting response surfaces therefore had a high maximum complexity and were ‘overfit’ in terms of statistical inference. However, here we were interested in visualizing a smoothed version of the data rather than drawing formal inferences.

Unfortunately the range of TN and TP values sampled varied too much between the different toxin groups to carry out the same analysis for the toxins as for the taxa. As such we were limited to examining the concentrations of total dissolved (particulate in the case of MC) toxin groups along simple TN and TP axes. We also correlate concentrations of the particulate fractions of toxin groups with the biovolumes of potential producing taxa.

## Results

### Lake Characteristics

Across the 102 lakes, summer total phosphorus ranged from 5 to 354 µg L^−1^ with the median within the range classified as eutrophic by the EU Water Framework Directive [58] and OECD [59] (Table1). As anticipated there was a significant positive correlation between logged TP and TN (r  = 0.63, p<0.001 on logged values) (Figure 1a), but there was still considerable variation in the TN:TP ratio which ranged widely from 2.8 to 96 with a median value of 22. TN rose less than proportionally with TP such that the mean TN:TP ratio declined with increasing nutrient enrichment, as illustrated by the fact that the dotted TN:TP isoline at the median value of 22 is below the major axis (solid red) at low TP, but above it at high TP (Figure 1a).

The sampled lakes included both polymictic and dimictic lakes with mean lake depth ranging from 1 to 23 m (Table 1). The median lake depth was 4.85 m, reflecting the preponderance of relatively shallow lakes in this region. Typically, shallow lakes have a lower TN:TP ratio but for this data set the relationship was weak (r^2^ = 0.07) and there was no relationship between the relative N vs. P enrichment score and depth (r^2^ = 0.01).

### Total Cyanobacterial Biovolume

Cyanobacterial maximum and median biovolume, as measured by the 90% and 50% quantiles respectively, both showed a positive relationship with total phosphorus (Figure 1c) and with total nitrogen concentrations (Figure 1d). However, the TN and TP relationships differed in shape: the TP–biovolume relationship was sigmoidal and flattened off at TP concentrations above approximately 50 µg L^−1^, perhaps indicating limitation by nitrogen or some other resource in high TP lakes, while the slope of the TN–biovolume relationship continued to increase over the entire range of TN values.

It is however difficult to interpret these relationships separately because of the strong correlation between TN and TP concentrations (Figure 1a). To address this we used contour plots of expected maximum biovolume to show whether maximum biovolumes were higher in lakes with high N enrichment relative to P or vice versa. We also calculated median enrichment scores weighted by biovolume to estimate the centre of the distribution of cyanobacteria relative to the average joint N and P and relative N vs. P axes. Figure 1b shows the 90% quantile of cyanobacterial biovolume on a filled contour plot where the intensity of the shading indicates the expected maximum biovolume for given combinations of TN and TP concentrations. The solid diagonal line shows the standardised major axis of the relationship between TN and TP. Dark green regions indicating biovolume concentrations above 30 mm^3^ L^−1^ lie in the top right quadrant of the plot, again indicating that maximum biovolumes were higher in lakes with high concentrations of both nitrogen and phosphorus. They are also found both above and below the SMA, indicating that observed maximum biovolumes were similar for lakes with high nitrogen relative to phosphorus and lakes with low nitrogen relative to phosphorus.

However, while maximum observed biovolumes of total cyanobacterial were similar on both sides of the major axis, the median total cyanobacteria N vs. P axis score was slightly positive, and bootstrapped 95% confidence intervals did not overlap with zero (0.08; 0.02–0.161), indicating that overall more cyanobacteria were found in high N vs. P lakes than in low N vs. P lakes.

### Distribution of Cyanobacterial Taxa

**Figure 2:**
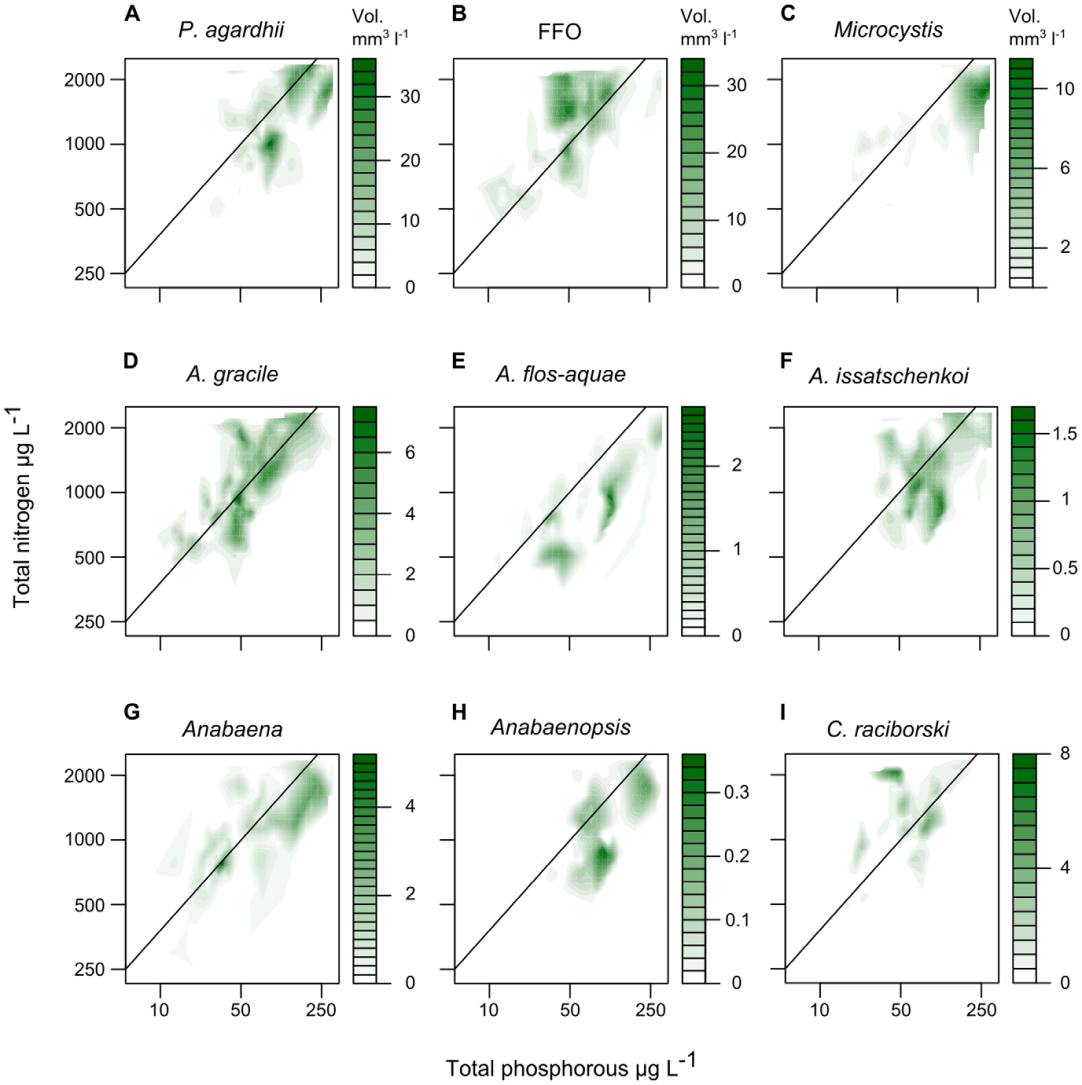
Filled contour plots of maximum biovolumes of nine cyanobacterial taxa on TN and TP axes. Filled contour plots showing, for 9 taxonomic groups of cyanobacteria, the fitted 90% quantile of biovolume as an estimate of the maximum expected biovolume of each taxa for a range of TN and TP concentrations.

**Figure 3:**
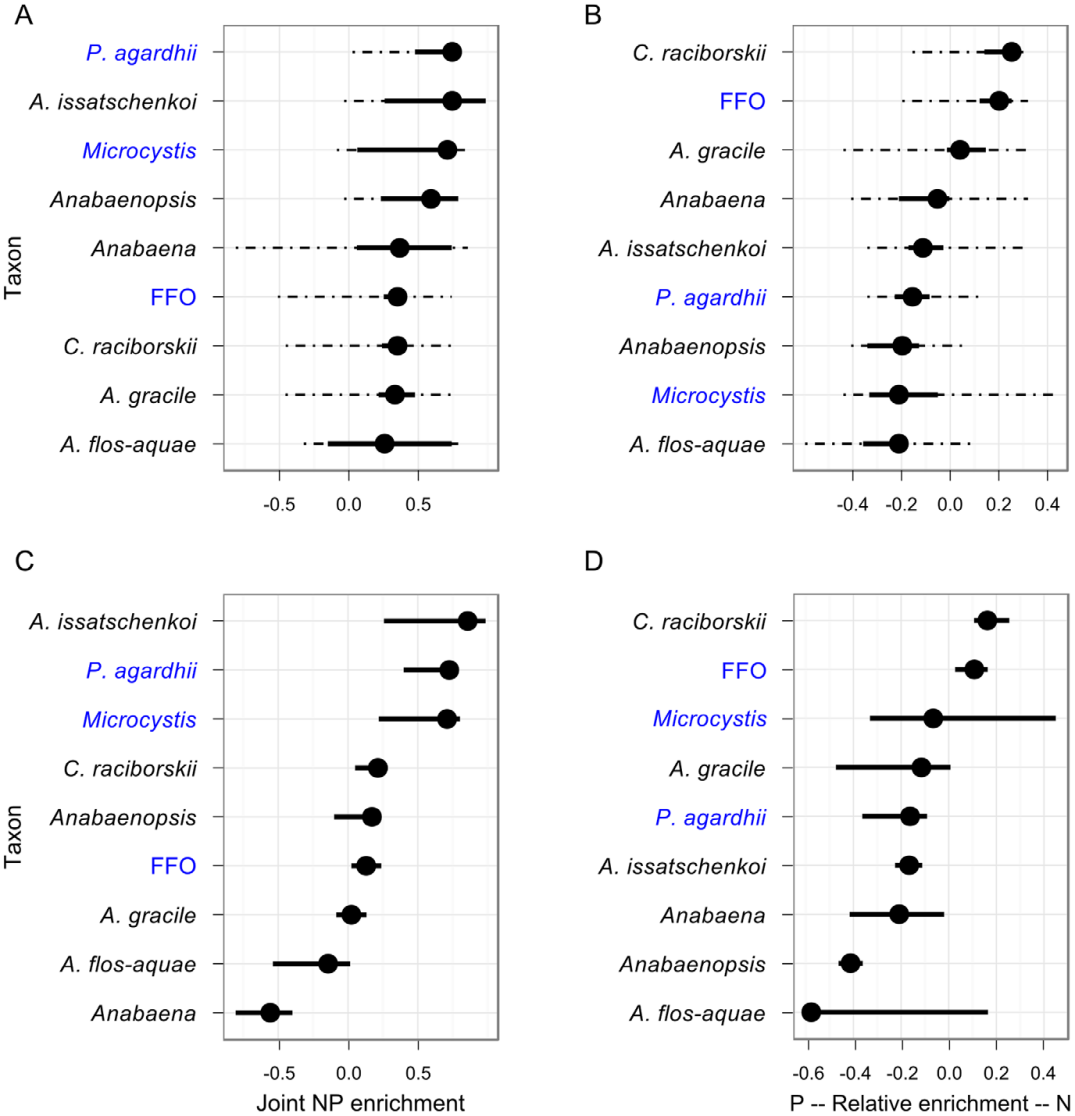
Location of nine cyanobacterial taxa on joint NP and relative N vs. P enrichment axes. The location along axes of joint NP enrichment (A) and relative N vs. P enrichment (B) of the expected centre of the distribution of the biovolume of 9 cyanobacteria taxa (points) plus the 95% confidence interval of this location (solid lines) plus the range in which 95% of the total biovolume is expected to be found. Names of N_2_ fixing taxa are in black, non-fixing taxa in blue. (C, D) as above except calculated for relative abundance. Points can be interpreted as the axis location where a taxon attains its highest relative abundance.

Differences in the distribution of the nine taxa with respect to joint NP and relative N vs. P enrichment are illustrated in Figures 2 and 3. Figure 2 shows fitted 90% quantiles of biovolume on TN and TP axes and indicates the locations of the larger observed biovolumes for each taxon. The location of shaded areas relative to the SMA (the diagonal line on each subplot) indicate whether the larger biovolumes for each taxa were found in lakes with relatively more (above) or less (below) nitrogen relative to phosphorus. Figure 3a–b shows the centre and limits of the distribution of the biovolume of each taxon on the joint NP and relative N vs. P axes. While Figure 3 mostly acts as a useful summary of the filled contour plots in Figure 2, correspondence is not necessarily 1:1; the centres of the distributions are normally close to the locations of highest biovolumes, but not always. Figure 3c–d shows where a taxon achieves its highest relative biovolume, relative to those of the other cyanobacteria.

Taxa did not differ greatly from each other in their distribution along the joint NP enrichment axis. All taxa had their distribution centres in lakes with above average joint NP scores (scores higher than zero in Figure 3a). *P. agardhii, A. issatchenkoi*, *Microcystis* and *Anabaenopsis* formed a group located at the highest levels of enrichment; with *Anabaena*, FFO, *C. raciborski, A. gracile*, and *A. flos-aquae* centred lower on the joint NP axis, but still in lakes with above average NP enrichment. The distinction between these two groups is clearer when considering the extents of their ranges (the dotted lines in Figure 3a): 95% of the biovolume of the first group of four taxa is found almost entirely above the average enrichment score, while much of the biovolume of the remaining 5 taxa is found below. This distinction is particularly clear for *Anabaena* and highlights the different pattern observed when considering absolute rather than relative abundances. *Anabaena* achieves its highest relative abundances in lakes with below average enrichment (joint NP scores of approximately -0.5 in Figure 3c), but it is actually more abundant in lakes with above average NP enrichment (joint NP scores of approximately 0.4 in Figure 3a), where it is joined by large biovolumes of other taxa and thus comprises a smaller fraction of the total biovolume.

In contrast to the joint NP axis, taxa were more widely distributed along the relative N vs. P axis. The distributions of *C.* *raciborski* and FFO were centred in lakes with greater N than P enrichment (Figure 3b) and the majority of their larger biovolumes were in high N, low P lakes (above the diagonal line in Figure 2 b, i). *A.gracile* was centred just above the middle of the N vs. P axis (Figure 3b) but was found at high biovolumes in both high-N-low-P and low-N-high-P lakes (above and below the diagonal line in Figure 2d). *Anabaena*, *A. issatschenkoi, P. agardhii*, *Anabaenopsis*, *Microcystis* and *A. flos-aquae* were all centred in low-N-high-P lakes (Figure 3b) and the majority of their larger biovolumes were also found in low-N-high-P lakes (below the SMA in Figure 2a, c, e, f, g, h).

The overall pattern observed for total cyanobacterial biovolume was mainly driven by two taxonomic groups: the fine-filamentous oscillatoriales (FFO), a heterogeneous group of non-fixing cyanobacteria; and the species *Planktotrix agarhdii*, also filamentous and non-fixing. Measured by total biovolume across all samples, FFO was the most abundant taxonomic grouping in the data set and was found at high biovolumes in lakes with high joint NP enrichment scores and with a strong tendency towards lakes relatively more enriched by N than P (above the SMA in Figure 2b, 3b); FFO had the second highest score on the N vs. P axis. *P.agarhdii* attained the highest biovolumes of any individual species and was found in lakes with the highest joint NP enrichment scores and with a tendency towards low-N-high-P environments.

Of the six N_2_-fixing taxa (Nostocales), four attained their highest biovolumes in low-N-high-P lakes. The exceptions were *A. gracile* and *C*. *raciborski*. *C*. *raciborski* in particular showed a clear tendency for developing its highest biovolumes in N-rich environments (Figure 2i) while the highest biovolumes of *Aphanizominon gracile* were found over a wide range of TN and a wide range of TP concentrations, corresponding to environments with both high-N-low-P and low-N-high-P concentrations.

### Cyanotoxins

**Figure 4:**
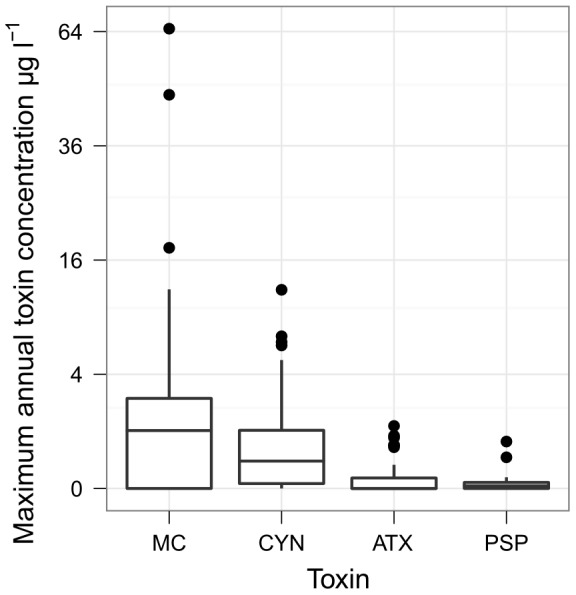
Maximum annual concentrations of four cyanotoxin groups. Boxplots of the maximum annual concentrations of four cyanotoxins sampled from north German lakes. MC concentrations come from 29 lake–summers at 13 lakes; CYN 56 lake–summers at 30 lakes; ATX 38 lake–summers at 14 lakes; and PSP 25 lake–summers at 13 lakes. Microcystin concentrations above 20 µg L^−1^ were all measured in 1995–96 and the samples were taken with a plankton net; all concentrations measured in subsequent years were lower than this.

**Figure 5:**
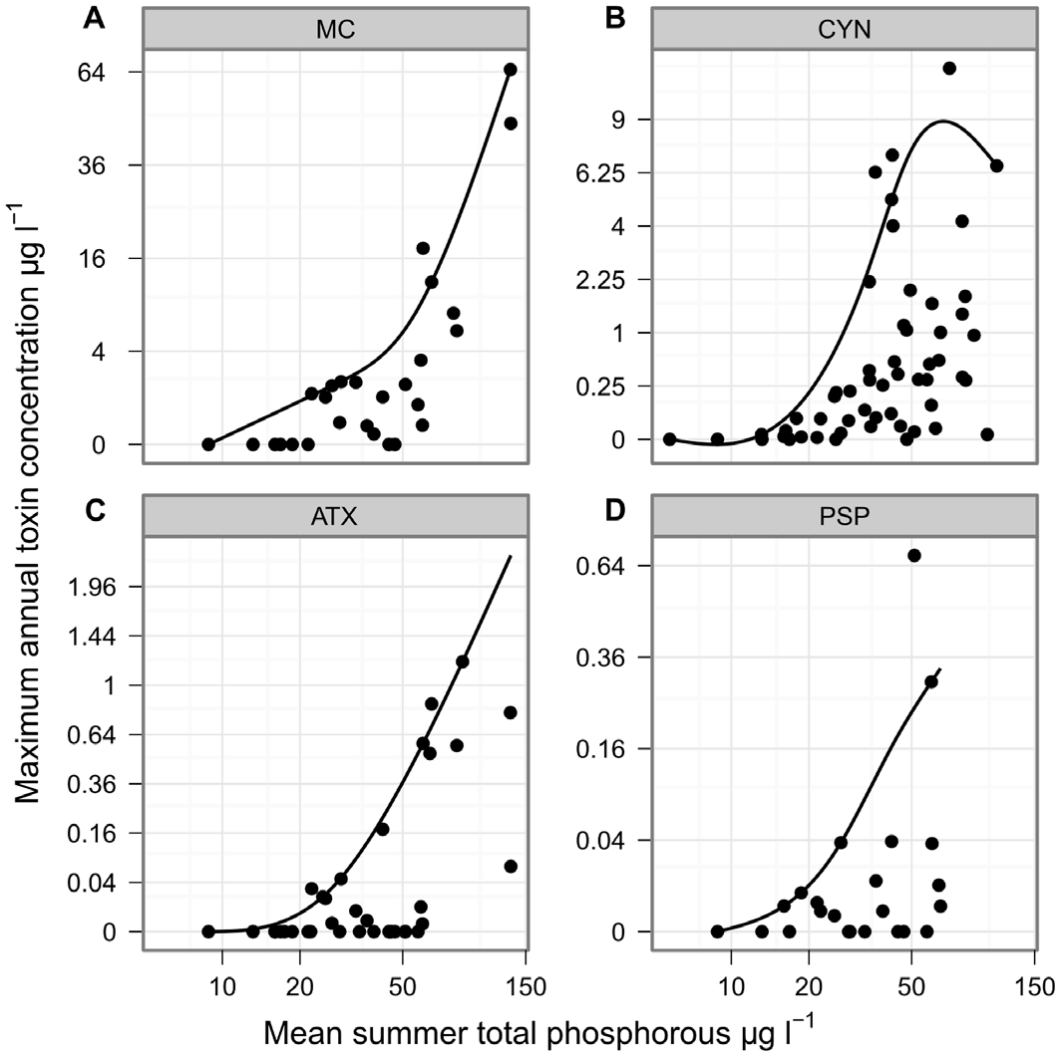
Maximum annual concentrations of four cyanotoxin groups against mean summer TP concentration. The relationship between annual maximum concentration of four cyanotoxins and mean summer TP concentration for lakes in Berlin and Brandenburg, Germany. The fitted line indicates the 90% quantile of toxin concentration as an estimate of the maximum expected concentration of toxin for a given TP concentration. Each point represents the mean TP and maximum toxin concentration for one lake–summer.

**Figure 6:**
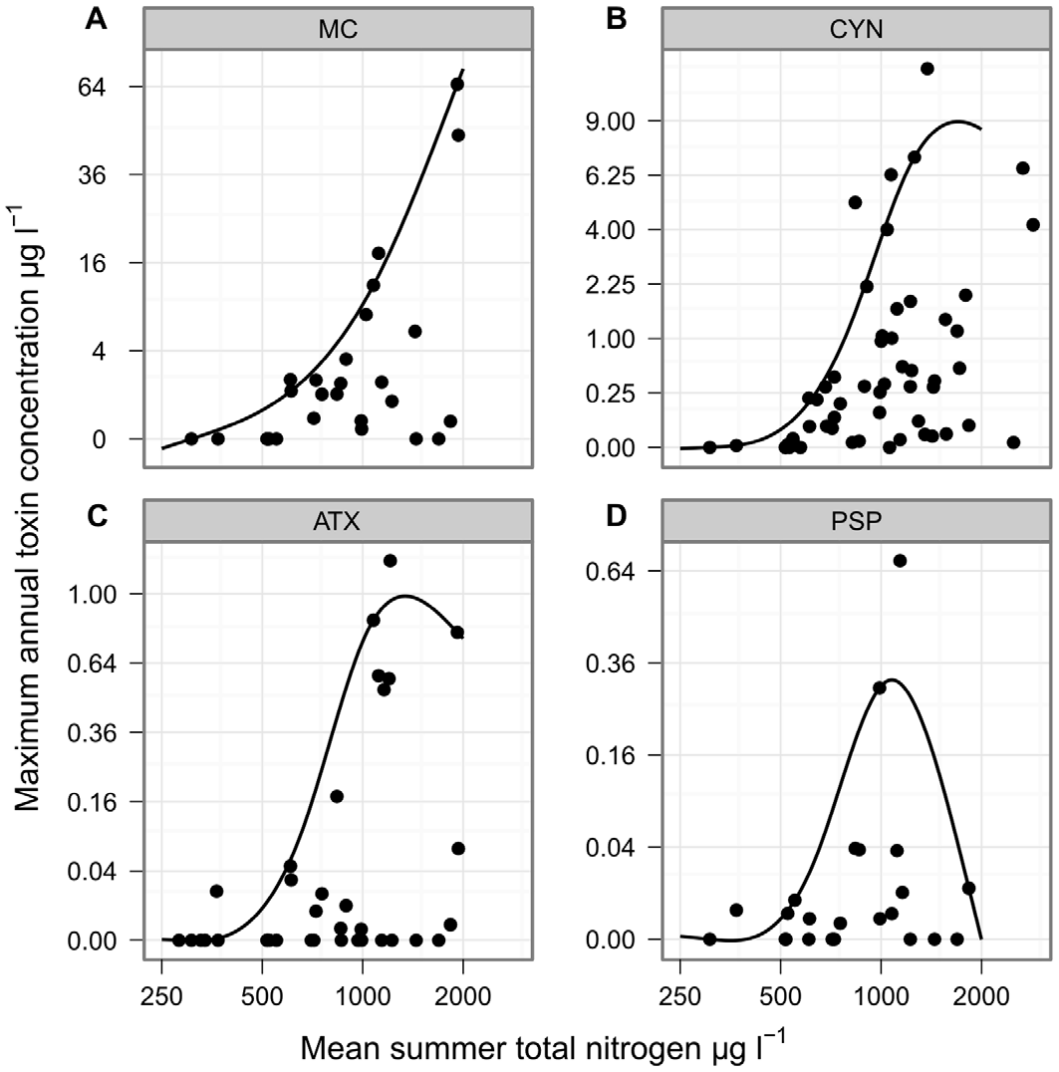
Maximum annual concentrations of four cyanotoxin groups against mean summer TN concentration. The relationship between annual maximum concentration of four cyanotoxins and mean summer TN concentration for lakes in Berlin and Brandenburg, Germany. The fitted line indicates the 90% quantile of toxin concentration as an estimate of the maximum expected concentration of toxin for a given TN concentration. Each point represents the mean TN and maximum toxin concentration for one lake–summer.

Cyanotoxin concentrations were measured in subsets of the lakes studied for cyanobacterial composition (Table 1). MC was detected at least once in 62%, CYN in 83%, ATX in 57% and PSP in 69% of the lakes tested. The highest concentration recorded for MC was 65 µg L^−1^, while those for CYN, ATX and PSP were much lower at 12.1, 1.2, and 0.7 µg L^−1^ respectively (Table 3; Figure 4). The expected maximum toxin concentrations, as determined by 90% quantiles, were highest at mid to high concentrations of TP and TN for all four groups of toxins (Figures 5 and 6).

**Table 3:** Occurrence and maximum concentrations of four cyanotoxins in Berlin and Brandenburg lakes.

	MC	CYN	ATX	PSP
No. lakes with non-zero toxin concentrations	8 of 13 (62%)	24 of 29 (83%)	8 of 14 (57%)	9 of 13 (69%)
No. lakes with toxin concentrations greater than 1 µg l^−1^	6 of 13 (46%)	9 of 29 (31%)	1 of 14 (7%)	0 of 13 (0%)
Maximum toxin concentration µg l^−1^	64.8	12.1	1.2	0.68

**Figure 7:**
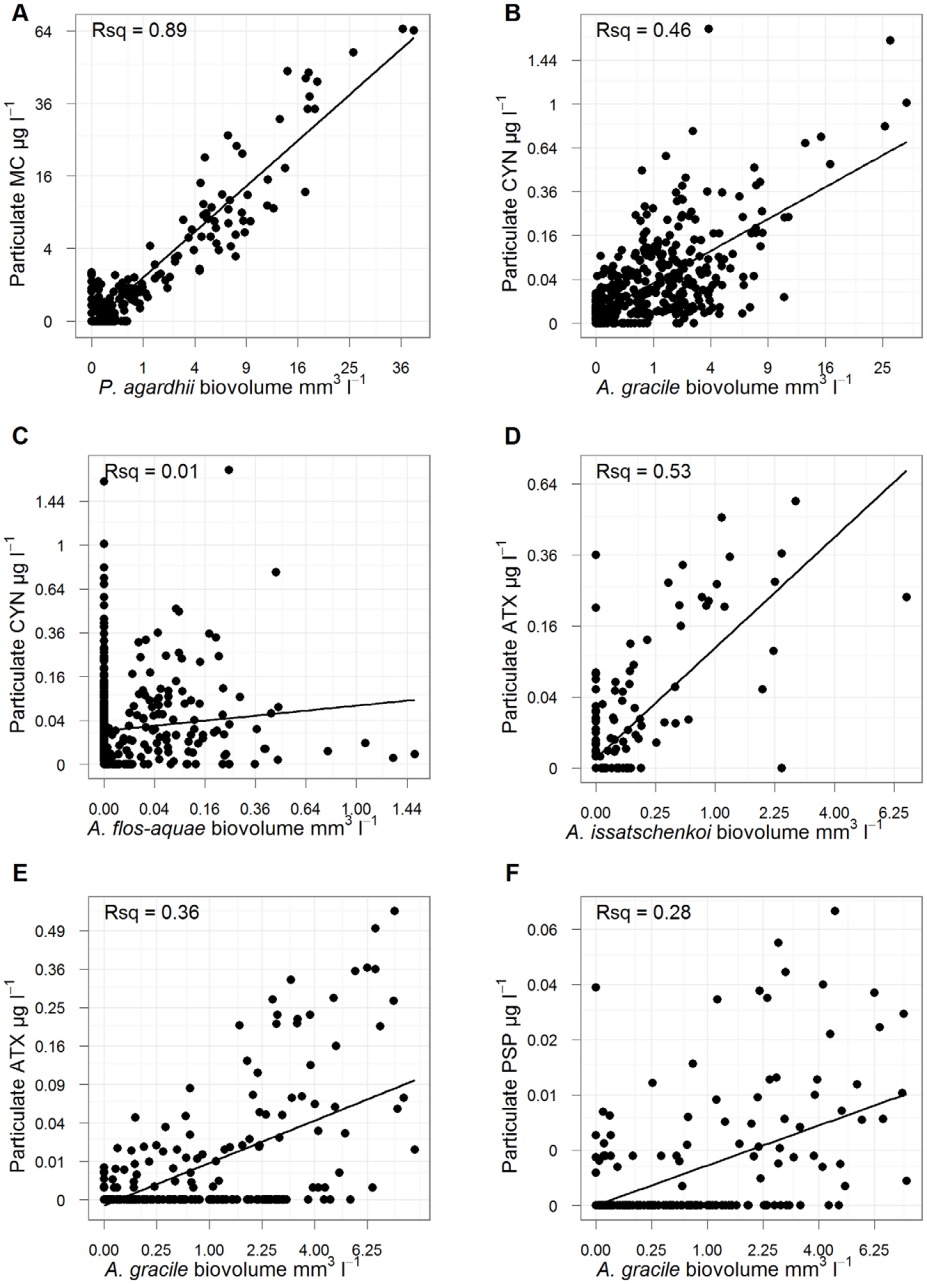
Correlations between the concentrations of four cyanotoxin groups and their potential producing taxa. Correlations between four cyanotoxin groups and previously identified potential producing cyanobacterial taxa. Correlations are between the particulate fractions of the toxin groups and biovolumes from individual sampling dates. For each subplot, points come from multiple years and lakes.

Particulate MC concentration correlated very closely with the biovolume of *P. agardhii* (Figure 7a; r^2^ = 0.89). Correlations with potentially producing taxa were much weaker for the three other toxin groups. Particulate ATX concentration correlated moderately well with *A. issatschenskoi* (Figure 7e; r^2^ = 0.53) but there were a number of relatively high ATX concentrations in the absence of any *A. issatschenskoi* biovolume; these perhaps can be explained by production by *A. gracile* which also correlated positively with particulate ATX (Figure 7f; r^2^ = 0.36). Particulate CYN was also correlated with *A. gracile* (Figure 7c; r^2^ = 0.46) but not with *A. flos-aquae* (Figure 7b; r^2^ = 0.01). Particulate PSP correlated poorly with its purported producer *A. gracile* (Figure 7b; r^2^ = 0.28).

## Discussion

### Distribution of Taxa

As anticipated, high nutrient concentrations were associated with high cyanobacterial biovolumes. We found a saturating relationship between logged TP concentration and cyanobacterial biovolume, as has been reported for chlorophyll-a [60]–[62] and cyanobacteria biomass elsewhere [26], indicating limitation by other factors in highly phosphorus enriched lakes. We found no such saturating relationship with TN and so nitrogen remains a candidate factor limiting cyanobacteria biovolume in high TP lakes. This is consistent with the view that an increasing proportion of lakes become N limited as trophic status increases [22].

Most abundant in our study were the non N_2_-fixing taxa FFO and *P. agardhii*. These two taxa showed contrasting responses to relative N vs. P enrichment. FFO was found preferentially in high-N-low-P lakes while the reverse was true for *P. agardhii*. In line with previous observations in this region, e.g. [30], [63], [64], *P. agardhii* showed a particularly strong response to phosphorus and attained its highest biovolumes, and the highest biovolumes of any taxa in this study, in those lakes with the very highest TP concentrations. The response of *P. agardhii* is of particular interest as it is the major microcystin producer in this region (see below) while FFO do not typically produce microcystins [47].

All nine cyanobacterial taxa attained their highest biovolumes in lakes with above average nutrient enrichment, but separated into two groups that differed in the range of their distribution along the joint NP enrichment axis (Figure 3a); almost no biovolume of *P.* *agardhii*, *A. issatschenkoi*, *Microcystis*, or *Anabaenopsis* was found in lakes with below average joint NP enrichment, while *Anabaena*, FFO, *C.* *raciborskii*, *A. gracile*, and *A. flos-aquae* had a significant portion of their biovolume in below average joint NP lakes. Of note is that biovolume maxima of several Nostocales taxa occurred at high nutrient enrichment levels (Figure 2) and they did not separate as a group from non-N_2_-fixing taxa along the joint NP enrichment axis (Figure 3a). These findings do not support previous observations that Nostocales biomass generally increases during the re-oligotrophication of lakes. Rather our results suggest that while many, but not all, Nostocales decline as a proportion of total cyanobacterial biovolume with increasing nutrient concentrations (Figure 3c), their absolute biovolumes increase as a function of nutrient enrichment, albeit reaching much lower maximums than those of other taxa. Note however that this was an analysis across lakes of differing trophic status and the re-oligotrophication process was not examined through time for individual lakes.

The separation of taxa maxima across the nitrogen versus phosphorus enrichment axis was more pronounced (Figure 3b); however, in contrast to the prevailing paradigm, Nostocales (N-fixing taxa) did not differ as a group from other cyanobacterial taxa in terms of their distribution in relatively N or P rich lakes. Although 4 of 6 Nostocales taxa were found in high P low N enriched lakes, the two most abundant were found in relatively high N low P enriched lakes. Thus the question arises, why do some N-fixing taxa occur at higher abundance in lakes with high nitrogen relative to phosphorus concentrations? Three possibilities should be considered here:

- Nostocales might be able to elevate ambient nitrogen levels through N_2_-fixation, as found by Vrede et al [25], to the extent that the lakes in which they are found have relatively high N concentrations. However, there is considerable evidence that Nostocales are not always able to compensate fully for N-deficiency, particularly in high productivity systems (reviewed in [17]).
- Environmental conditions may constrain N-fixation. Nitrogen fixation is a very energy demanding process and therefore Nostocales generally require high light intensities to maintain growth and N-fixation at the same time [65], [66]. Indeed it has been suggested that N-fixers are not able to become dominant in turbid eutrophic lakes [67] or only as long as enough light is penetrating into the mixed water during early summer [47]. The availability of micronutrients such as iron or molybdenum may also limit nitrogen fixation [68].
- The intrinsic capacity of individual nostocalean species to fix nitrogen may differ, and not all species may be able to overcome N-deficiency. For example, the N-fixation rate per heterocyst of several *Aphanizomenon flos-aquae* strains is three times higher than those of several *Anabaena* strains 69. Our data suggest that *C. raciborskii* and *A. gracile* are weak N-fixers. However, for these two species, as well as many other species, data about their N-fixation capacity are lacking.

More information is needed about the intrinsic capacity for N-fixation of different species, as well as *in situ* N-fixation rates under typical environmental conditions, in order to fully answer the question as to why some Nostocales occur at high TN relative to TP concentrations. To date, many studies that focused on the occurrence of Nostocales at different N:P ratios, and their capability to compensate for N-deficiency, have treated Nostocales as a single response group, e.g. [70], or were carried out in lakes where *C. raciborskii*, considered a subtropical potentially invasive species, is not present or was not present at that time [25], [71].

### Cyanotoxins

A question of major concern is whether the occurrence and concentrations of cyanotoxins can be predicted from the abundances of their producing taxa. Identifying the producers of cyanotoxins is difficult because each toxin group can have a number of different producing taxa, each taxa can have toxigenic and non-toxigenic strains that may or may not co-occur, and the cellular toxin content of specific toxigenic strains can itself vary due to environmental conditions [72].

In our study, MC concentrations were strongly correlated with the biovolume of *P. agardhii* (Figure 7a). MC is also known to be produced by several *Microcystis* and *Anabaena* species [6], [73], and some of the remaining variability might be due to minor amounts of MC production by these taxa, but to a great extent MC concentrations can be explained by the biovolume of *P. agardhii* in these lakes. Therefore high MC concentrations occur in the same circumstances as high biovolumes of *P. agardhii*, which, in this region, are high nutrient enrichment levels, particularly high TP concentrations (Figure 2a and 3a). In other regions MC has been found at high concentrations in lakes of relatively low trophic state, e.g. in Scandanavia [51], while in the American midwest, Graham [42] found that MC concentration declined at concentrations of TN and TP greater than those in our region.

In contrast to MC, concentrations of CYN, ATX and PSP were more weakly correlated with the studied taxa. Various different Nostocales taxa are known producers of these three toxins (reviews in [35], [36], [74]) but there seems to be geographical differences in the occurrence of toxigenic strains. For example CYN producing strains of *C. raciborskii* have been isolated from many tropical freshwaters while none of the strains isolated from European waters were found to produce CYN [75]. So far in this region, strains of *A. flos-aquae* have been identified as CYN producers [33]; however the stronger correlation we found suggests *A. gracile* as the main CYN producer here. *A. issatschenkoii* has been shown to produce ATX [35] and the correlation seen here suggest that it is at least one of the main producers in this region. Ballot et al [36] identified PSP producing strains of *A. gracile* but we found only a weak correlation with the latter.

This poorer correlation between CYN, ATX and PSP and their potential producers may be because not all producers have yet been identified or because the share of toxigenic strains in populations of these taxa is much more variable in time and space than are the share of MC producing strains in populations of *P.agardhii*. In fact the share of CYN, ATX or PSP producing strains in the studies of Preußel et al. [33] and Ballot et al. [35], [36] were very low compared to the typical share of MC producing *Planktothrix* strains, e.g. [37].

Annual maximum concentrations of all four toxin groups increased with nutrient concentration along the ranges observed here. This matched well with the pattern observed in the absolute biovolumes of taxa: all taxa were more abundant in nutrient rich lakes, even those such as *Anabaena* that are sometimes associated with oligotrophic lakes due to their higher relative abundances in these conditions. However, we expect that concentrations of CYN, ATX and PSP will be lower than that of MC in lakes more nutrient rich than those for which we had toxin data, because at the upper end of our nutrient enrichment gradient the biovolumes of their potential producers are much lower than that of MC producing *P. agardhii*.

Although at low nutrient concentrations biovolumes of potential CYN, ATX, and PSP producing Nostocales were sometimes higher than that of MC producing *P. agardhii* (Figure 2), this was not mirrored by higher concentrations of nostocalean toxins than of MC. However, fewer oligo- and mesotrophic lakes were included in this dataset because these lake types are rare in the study region. Therefore, caution should be exercised regarding Nostocales toxins in nutrient poor lakes. In an extreme case, toxigenic strains might dominate within a population of a Nostocales species, resulting in relatively high toxin concentrations at relatively low population size. High MC concentrations have been observed in nutrient poor deep dimictic lakes that are dominated by other *Planktothrix* species, e.g. *P. rubescens,* a very potent MC producer [76], or different strains of *P. agardhii*, which can behave quite differently in other regions (e.g. Scandinavia [51]).

### Conclusion

Our data clearly show that cyanobacteria have diverse responses to varying nitrogen vs. phosphorus enrichment and should not be treated as a single group when considering the effects of nutrient loading on phytoplankton community structure. This is of particular importance when considering cyanotoxin occurrence. Our data do not generally support the view that N-fixing taxa are more abundant in lakes with relatively more P than N enrichment, because the two most abundant Nostocalean species occurred in high N relative to P lakes. Despite the strong experimental and statistical evidence for the role of phosphorus in explaining cyanobacterial biomass and dominance as lakes become more eutrophic, there is a significant effect of N in explaining differences in species composition between lakes. Given additional data we expect this pattern to be reflected in the distribution of cyanotoxins.
